# Functional characterization of TBR1 variants in neurodevelopmental disorder

**DOI:** 10.1038/s41598-018-32053-6

**Published:** 2018-09-24

**Authors:** Joery den Hoed, Elliot Sollis, Hanka Venselaar, Sara B. Estruch, Pelagia Deriziotis, Simon E. Fisher

**Affiliations:** 1 Language and Genetics Department, Max Planck Institute for Psycholinguistics, 6525 XD Nijmegen, The Netherlands; 2 Centre for Molecular and Biomolecular Informatics, Radboud University Nijmegen Medical Centre, 6525 GA Nijmegen, The Netherlands; 3 Donders Institute for Brain, Cognition and Behaviour, 6525 EN Nijmegen, The Netherlands

## Abstract

Recurrent *de novo* variants in the TBR1 transcription factor are implicated in the etiology of sporadic autism spectrum disorders (ASD). Disruptions include missense variants located in the T-box DNA-binding domain and previous work has demonstrated that they disrupt TBR1 protein function. Recent screens of thousands of simplex families with sporadic ASD cases uncovered additional T-box variants in TBR1 but their etiological relevance is unclear. We performed detailed functional analyses of *de novo* missense TBR1 variants found in the T-box of ASD cases, assessing many aspects of protein function, including subcellular localization, transcriptional activity and protein-interactions. Only two of the three tested variants severely disrupted TBR1 protein function, despite *in silico* predictions that all would be deleterious. Furthermore, we characterized a putative interaction with BCL11A, a transcription factor that was recently implicated in a neurodevelopmental syndrome involving developmental delay and language deficits. Our findings enhance understanding of molecular functions of TBR1, as well as highlighting the importance of functional testing of variants that emerge from next-generation sequencing, to decipher their contributions to neurodevelopmental disorders like ASD.

## Introduction

*TBR1* (T-box brain, 1; OMIM *604616) encodes a neuron-specific transcription factor of the T-box family^1^. The TBR1 protein is highly expressed in the deep layers of the cortex, where it is involved in differentiation of subsets of projection neurons^2–4^. The importance of this transcription factor in neurodevelopment is evident from studies of mice that lack the *Tbr1* gene^5,6^. Homozygous mutants show abnormalities in cortical lamination and die shortly after birth^5,6^, whereas heterozygous mutants display reduced inter- and intra-amygdalar connections and display autistic-like behaviors^7^.

Recently, TBR1 has emerged as a master regulator of transcription in autism spectrum disorders (ASD) by regulating the expression of ASD-related genes that are critical for cortical development, including *RELN*, *GRIN2B* and *AUTS2*^2,8–10^. Furthermore, *TBR1* is one of several genes reported to be recurrently disrupted in unrelated cases of sporadic ASD. Heterozygous variants identified in *TBR1* include whole-gene deletions^11,12^, frameshifting and truncating variants^13^, and missense variants^13–19^. The majority of the reported *TBR1* variants occur *de novo*, consistent with the severe phenotypes described for the affected cases. The detection of whole-gene deletions suggests that the main pathogenic mechanism is haploinsufficiency. This is supported by investigations of the functional effects of missense and truncating *TBR1* variants in cell model systems^18^. However, TBR1 forms homodimers and experimental analyses have indicated that some heterozygous variants might exert an additional dominant-negative effect by interfering with the function of the normal protein^18^.

Investigations into TBR1 biology have pointed towards molecular links between clinically-distinct neurodevelopmental phenotypes. TBR1 interacts with the forkhead transcription factors FOXP1 (OMIM *605515) and FOXP2 (OMIM *605317)^18,20^, both of which are implicated in neurodevelopmental disorders characterized by speech and language impairment, of differing severity and specificity (OMIM #613670 and #602081, respectively)^21–23^. Furthermore, TBR1 interacts with the membrane-associated guanylate kinase CASK (OMIM *300172), which functions as a TBR1 co-activator and is implicated in X-linked intellectual disability and ASD (OMIM #300749)^24,25^. Recent studies identified the BCL11A transcription factor (OMIM *606557) as a direct regulator of *TBR1* expression in the cortex^26^. Like TBR1, BCL11A is found in deep cortical layers where it plays an essential role in establishing subsets of projection neurons in the developing cerebral cortex^26,27^ and *BCL11A* variants have been reported in cases of ID and autistic features (OMIM #617101)^28^. TBR1 and BCL11A co-localize in a subset of cortical layers^26^, where they may interact to regulate gene networks important for neurodevelopment.

Here we report detailed functional characterization of three *de novo TBR1* missense variants that were identified in ASD/ID cases by next-generation sequencing^15–17^. All three variants are found within the T-box DNA-binding domain and are predicted to be deleterious to protein function, but none have been previously tested for their functional effects. We assessed their impact on subcellular localization, transcriptional activity and protein interactions and show that out of the three variants, only two abolish protein functions. The third missense variant did not have an effect on any of the assays performed in this study. Furthermore, we show that TBR1 interacts with BCL11A and investigate the effect of etiological variants on this interaction. Overall, our results shed new light on the pathological mechanisms conferred by *TBR1* variants in cases of ASD/ID and provide further insight into the molecular functions of this important neural transcription factor.

## Results

### *De novo TBR1* variants in sporadic ASD and ID cases

**Figure 1:**
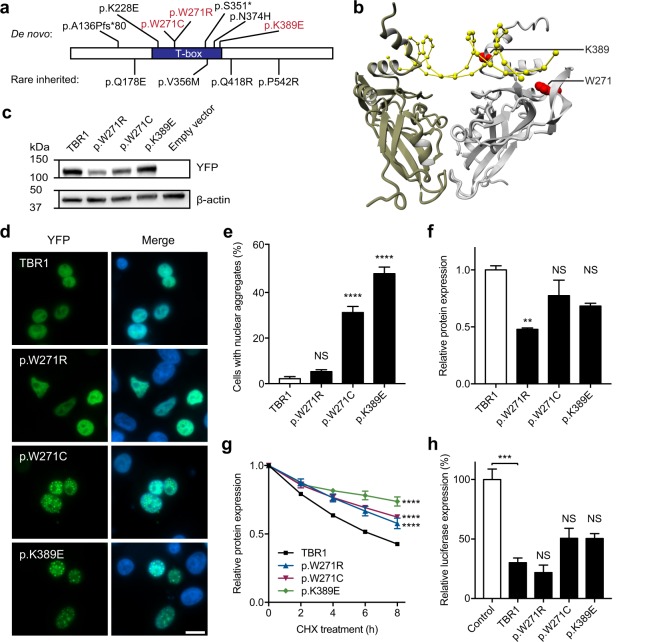
Functional characterization of *de novo* missense *TBR1* variants. (**a**) Schematic representation of the TBR1 protein indicating *de novo* and rare inherited variants found in cases of sporadic ASD. The three missense variants being functionally validated in this study are shown in red. (**b**) Three-dimensional homology model of the TBR1 T-box dimer in interaction with DNA, based on the PDB 2X6U protein structure. Monomers are shown in gold and grey, and the DNA is depicted in yellow. The mutated residues that were functionally validated in this study are shown in red. (**c**) Immunoblot of whole-cell lysates expressing YFP-tagged TBR1 variants probed with anti-EGFP antibody. Expected molecular weight for all variants is ~100 kDa. The blot was stripped and re-probed for β-actin, to ensure equal protein loading. (**d**) Direct fluorescence imaging of cells expressing YFP-TBR1 fusion proteins (green). Nuclei were stained with Hoescht 33342 (blue). Scale bar = 10 µm. (**e**) Quantification of nuclear aggregation of YFP-TBR1 fusion proteins. The bar chart displays the mean percentage of cells containing protein aggregates (three independent experiments performed in duplicate ± S.E.M., ****P < 0.0001, NS: not significant, one-way ANOVA and *post-hoc* Tukey’s test). In each experiment ~300–400 YFP-expressing cells were used for scoring. (**f**) Relative expression levels of TBR1 protein variants in live cells as measured by YFP fluorescence (mean of three independent experiments performed in triplicate ± S.E.M., **P < 0.01, NS: not significant, one-way ANOVA and *post-hoc* Tukey’s test). (**g**) Results of assay for protein degradation of TBR1 variants, using cycloheximide (CHX) to arrest protein synthesis. Values represent the mean expression levels of TBR1 protein variants in live cells as measured by YFP fluorescence and expressed relative to the 0 h time point (three independent experiments performed in triplicate ± S.E.M., ****P < 0.0001 at 8 h time point compared to WT TBR1, repeated measures two-way ANOVA and *post-hoc* Tukey’s test). (**h**) Luciferase reporter assay using a reporter construct containing a conserved element near *Fezf2*. Values are expressed relative to the control and represent the mean ± S.E.M. of three independent experiments performed in triplicate (***P < 0.001 when compared to control, NS: not significant when compared to WT TBR1, one-way ANOVA and *post-hoc* Tukey’s test).

Our prior molecular investigations of *de novo* truncating and missense *TBR1* variants uncovered by whole-exome sequencing of sporadic ASD demonstrated severe effects on protein function^18^. Since then, next-generation sequencing studies have reported additional *de novo TBR1* variants, including three missense variants (p.W271R, p.W271C and p.K389E) that are located in the T-box domain^15–17^ (Fig. 1a and Table 1). The p.W271R variant was found in a study of 41 probands with moderate to severe ID^15^. The patient carrying this variant was also diagnosed with autism and described as non-verbal and unable to understand simple commands. A variant affecting the same tryptophan residue but resulting in a cysteine substitution (p.W271C) was identified in a proband in a large cohort of 3,871 sporadic ASD cases^16^. The third variant, p.K389E, was discovered in a proband with ASD and ID in a study targeting candidate genes already implicated in ASD^17^. All three variants are predicted to be deleterious based on Combined Annotation Dependent Depletion (CADD) scores^29^, but to our knowledge there is no available experimental evidence regarding their effects on function. Three-dimensional modeling of the TBR1 T-box bound to DNA, based on structural data of the T-box domain of the protein family member TBX5, and subsequent mutation analysis of these variants demonstrated disturbance of direct non-specific binding to the DNA backbone at the minor groove (p.K389E), or destabilization of the core structure of the T-box domain (p.W271R and p.W271C) (Fig. 1b and Supplementary Notes). Therefore, we sought to extensively characterize the consequences of these T-box variants on diverse aspects of protein function.

**Table 1:** Description of *de novo TBR1* variants identified in sporadic ASD and ID cases.

Study	Proband ID	gDNA (hg19)	cDNA (NM_006593.3)	Residue change	PolyPhen-2 score	CADD v.1.3
Hamdan *et al*. (ref.^15^)	121.83	Chr2: 162274305T > C	c.811T > C	p.W271R	Probably damaging	25.7
De Rubeis *et al*. (ref.^16^)	09C86693	Chr2: 162274307G > T	c.813G > T	p.W271C	Probably damaging	27.5
O’Roak *et al*. (ref.^17^)	220-9833-201	Chr2: 162276743A > G	c.1165A > G	p.K389E	Probably damaging	27.1

### Expression, localization, protein stability and transrepression of TBR1 variants

The three T-box TBR1 variants as well as the wild type (WT) protein were expressed as fusions to YFP in HEK293 cells. Immunoblotting showed that all constructs produced proteins at the expected molecular weight (~100 kDa) (Fig. 1c). Direct fluorescence imaging showed that WT TBR1 was evenly distributed in the nucleus, but excluded from the nucleoli, as previously reported (Fig. 1d)^18^. The p.W271C and p.K389E variants also localized to the nucleus, but formed large aggregates in 30–50% of the cells (Fig. 1d and e). Although the p.W271R variant affects the same residue as p.W271C, p.W271R showed a normal localization pattern similar to WT TBR1 (Fig. 1d and e). Western blotting suggested that this variant may be expressed at lower levels compared to WT TBR1 (Fig. 1c). Quantification of protein expression levels of all variants in live cells based on YFP as well as mCherry fluorescence intensity confirmed that the expression level of the p.W271R variant was significantly lower compared to WT TBR1 (Fig. 1f and Supp. Figure 1a).

To test for differences in protein stability, cycloheximide was added to cells expressing either WT TBR1 or TBR1 variants in order to arrest protein synthesis, and the decrease in protein over time was monitored in live cells based on YFP fluorescence intensity. For WT TBR1, the amount of protein dropped to approximately 40% of starting levels after 8 h incubation with cycloheximide. The p.W271R, p.W271C and p.K389E variants appeared to be more stable (Fig. 1g). Two previously studied *de novo* TBR1 missense variants (p.K228E and p.N374H) that form nuclear aggregates^18^, similar to p.W271C and p.K389E, were also more stable than WT TBR1 (Supp. Figure 1b). In contrast, rare inherited TBR1 variants, that do not affect TBR1 protein function in cell-based assays in a prior study and do not form protein aggregates^18^, showed no difference in the rate of degradation compared to WT TBR1 (Supp. Figure 1b).

To examine the effects of the variants on the ability of TBR1 to regulate transcription we performed luciferase reporter assays. We used a luciferase reporter construct containing a conserved consensus element found near murine *Fezf2*, which has been shown to be directly regulated by Tbr1^3,18^. As previously reported, WT TBR1 repressed luciferase activity (*P* < 0.001) (Fig. 1h). All three TBR1 variants retained the capacity to repress luciferase activity in this assay (Fig. 1h). Similar findings have been reported for two additional *de novo* variants located in the T-box domain (p.K228E and p.N374H)^18^.

### TBR1 variants disrupt CASK translocation to the nucleus

**Figure 2:**
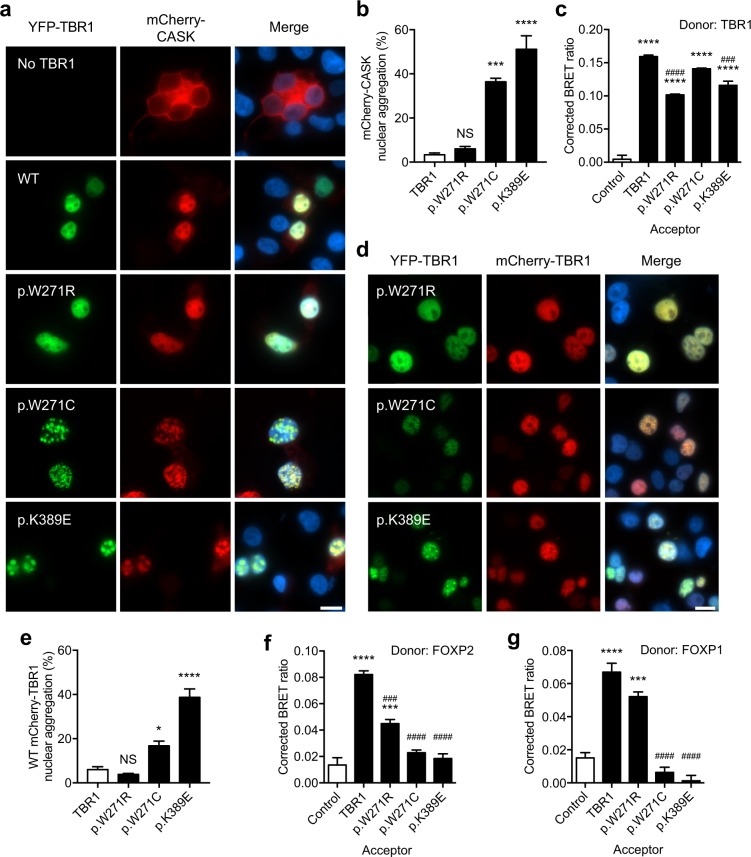
Effect of TBR1 variants on known protein interactions. (**a**) Fluorescence micrographs of cells co-transfected with TBR1 variants and CASK. TBR1 variants are fused to YFP (green, left panel) and CASK is fused to mCherry (red, middle panel). Nuclei were stained with Hoescht 33342 (blue). Scale bar = 10 µm. (**b**) Quantification of nuclear aggregation of mCherry-CASK when co-expressed with YFP-TBR1 variants. In each independent experiment ~100–250 cells were scored. (**c**) BRET assay for interaction between WT TBR1 and TBR1 variants in live cells. (**d**) Fluorescence micrographs of cells co-expressing WT TBR1 fused to mCherry (red; middle panel) and TBR1 variants fused to YFP (green; left panel). Nuclei were stained with Hoescht 33342 (blue). Scale bar = 10 µm. (**e**) Quantification of nuclear aggregation of WT mCherry-TBR1 when co-expressed with YFP-TBR1 variants. In each independent experiment ~300–600 cells were scored. (**f**) BRET assay for interaction between FOXP2 and TBR1 variants. (**g**) BRET assay for interaction between FOXP1 and TBR1 variants. In (**b**) and (**e**) the bars represent the mean percentage of cells containing protein aggregates, determined from three independent experiments performed in duplicate (*P < 0.05, ***P < 0.001, ****P < 0.0001, NS: not significant, one-way ANOVA and *post-hoc* Tukey’s test). In (**c**), (**f**) and (**g**) bars represent the corrected mean BRET ratios ± S.E.M. of one representative experiment performed in triplicate (***P < 0.001, ****P < 0.0001 when compared to NLS-control, ^###^*P* < 0.001, ^####^*P* < 0.0001 when compared to WT TBR1, one-way ANOVA and *post-hoc* Tukey’s test).

One of the few proteins that is known to interact with TBR1 is the membrane-associated guanylate kinase CASK^8,18^. CASK is important for neural development and synaptic function^30^. Heterozygous variants disrupting *CASK* have been found in cases of severe ID and ASD^24,25^. The interaction with TBR1 translocates CASK from the cytoplasm to the nucleus^8,18^, where it functions as a cofactor in the regulation of TBR1 target genes such as *RELN* and *GRIN2B*^8,31^. We have previously shown that *de novo* truncating and missense *TBR1* variants lead to abnormal CASK translocation to the nucleus^18^. To assess if the three T-box variants studied here - p.W271C, p.W271R and p.K389E - affect the TBR1-CASK interaction, we co-transfected HEK293 cells with CASK fused to mCherry and TBR1 variants fused to YFP. Similar to WT TBR1, all three variants co-localized with CASK in the nucleus (Fig. 2a). Two of the three variants, p.W271C and p.K389E, co-localized with CASK in nuclear aggregates in 40–50% of the cells that expressed nuclear CASK (Fig. 2a and b), as previously observed for other missense *de novo* TBR1 variants that are found in the T-box domain^18^.

### TBR1 variants affect known protein interactions

Similar to other T-box transcription factors, TBR1 can form homodimers^18^. To investigate whether the three TBR1 variants affect self-association and/or interaction with WT TBR1, we employed the Bioluminescence Resonance Energy Transfer (BRET) assay which monitors protein interactions in live cells^32^. All three variants retained the ability to self-associate and interact with WT protein (Fig. 2c and Supp. Figure 1c). In our protein homology model of the TBR1 T-box bound to DNA, we found four potential conformations for homodimerization (Supplementary Notes). Indeed, the W271 and K389 residues are not located at the dimerization interfaces in any of these conformations, suggesting that they do not play a direct role in homodimerization. When co-expressed with WT TBR1, two of the variants - p.W271C and p.K389E - resulted in mislocalization of the WT protein in nuclear aggregates in 20–40% of the cells (Fig. 2d and Fig. 2e). A similar effect was previously observed for other missense TBR1 variants (p.K228E and p.N374H) that form nuclear aggregates^18^. It is therefore possible, that in patient cells, these *de novo* missense variants exert a dominant-negative effect on the function of WT TBR1.

Beyond CASK, TBR1 is known to interact with the forkhead box transcription factors FOXP2 and FOXP1^18,20^. Disruptions in *FOXP2* cause a severe speech and language disorder^21^, whereas *FOXP1* variants have been implicated in syndromic ID^22,23^. Prior work has demonstrated that etiological *TBR1* variants abolish the TBR1-FOXP2 interaction^18^ and that pathogenic *FOXP1* variants abolish the TBR1-FOXP1 interaction^20^. Together, these findings point towards molecular links between distinct neurodevelopmental phenotypes that involve language deficits. Here, using the BRET assay, we found that the p.W271C and p.K389E variants also disrupted the TBR1-FOXP2 and TBR1-FOXP1 interactions (Fig. 2f and g). Our results are in line with previous work showing that the T-box domain is important for mediating the TBR1-FOXP2 interaction^18^. Furthermore, the lack of interaction that we observed cannot be explained by aberrant subcellular localization, as both p.W271C and p.K389E localize to the nucleus (Supp. Figure 1d and e). Intriguingly, the p.W271R variant behaved similarly to WT TBR1 by retaining the ability to interact with FOXP2 and FOXP1 (Fig. 2f and g).

### TBR1 interacts with BCL11A

**Figure 3:**
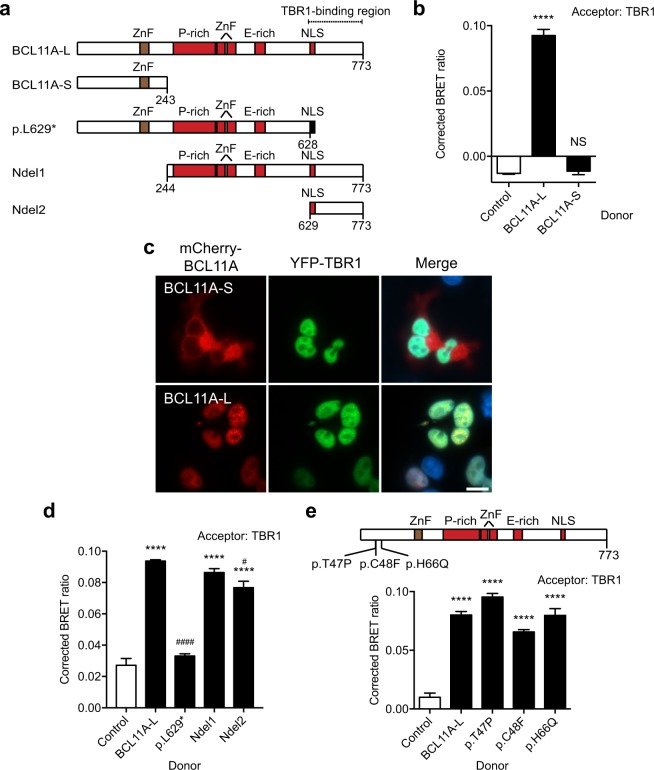
TBR1 interacts with BCL11A-L. (**a**) Schematic of recombinant BCL11A proteins used in BRET assays. BCL11A-L and BCL11A-S represent naturally occurring isoforms, other constructs are synthetic. The two DNA-binding zinc finger (ZnF) domains are shaded red; the non-DNA-binding ZnF is shaded brown. Proline (P)- and glutamate (E)-rich regions are also shown as well as a putative NLS spanning residues 629–642 (based on cNLS mapper^49^). An SV40 Large T-antigen NLS appended to the C-terminus of the p.L629* variant is shaded black. (**b**) BRET assay for interaction between TBR1 and naturally-occurring BCL11A isoforms. (**c**) Fluorescence micrographs of cells co-expressing BCL11A variants fused to mCherry (red; left panel) and WT TBR1 fused to YFP (green; middle panel). Nuclei were stained with Hoescht 33342 (blue). Scale bar = 10 µm. (**d**) BRET assay with TBR1 and synthetic BCL11A constructs. (**e**) BRET assay with TBR1 and *de novo* missense BCL11A variants found in cases of Dias-Logan syndrome. In (**b**), (**d**) and (**e**) bars represent the corrected mean BRET ratio ± S.E.M. of one representative experiment performed in triplicate (in (**b**) ****P < 0.0001, NS: not significant when compared to NLS-control, in (**d**) and (**e**) ****P < 0.0001 when compared to NLS-control, ^####^*P* < 0.0001, ^#^*P* < 0.05 when compared to WT BCL11A, one-way ANOVA and *post-hoc* Tukey’s test).

Several lines of evidence suggest that the BCL11A transcription factor may interact with TBR1. The two proteins have been shown to co-localize in deep layers of the cortex^26^ and they both interact with CASK^8,33^. We used the BRET assay to investigate the interaction between TBR1 and two naturally-occurring BCL11A isoforms expressed in the human brain: BCL11A-S (243 amino acids) and BCL11A-L (773 amino acids) (Fig. 3a)^34^. BCL11A-L is nuclear and interacts with TBR1 (Fig. 3b and c). In contrast, we did not detect a TBR1-BCL11A-S interaction (Fig. 3b). When BCL11A-L is absent, BCL11A-S localizes to the cytoplasm, which may explain the observed lack of interaction with TBR1 (Fig. 3c). An alternative explanation may be that the region important for mediating the TBR1-BCL11A interaction lies downstream of the N-terminal region of BCL11A.

To test this hypothesis, we generated truncated BCL11A proteins (Fig. 3a). We first created two N-terminal deletions, spanning residues 244–773 and 629–773 (Fig. 3a and Supp. Figure 2a). Direct fluorescence imaging of mCherry-fusion proteins indicated that these N-terminal deletions localize to the nucleus, similar to WT BCL11A (Supp. Figure 2b). BRET assays demonstrated that both N-terminally-truncated variants retained the ability to interact with TBR1, suggesting that the first 628 amino acids may not be required for the TBR1-BCL11A interaction (Fig. 3d). Accordingly, we found that *de novo* missense BCL11A variants (p.T47P, p.C48F and p.H66Q) identified in cases of global developmental delay and moderate to severe ID^28^ did not affect TBR1-BCL11A interaction (Fig. 3e and Supp. Figure 2c).

To confirm the BCL11A region important for interaction with TBR1, we created a C-terminal truncation, p.L629*, appended with a nuclear localization signal (NLS); this variant lacks the final 145 residues in BCL11A (Fig. 3a and Supp. Figure 2a). BRET assays showed that this BCL11A p.L629* variant abolished the TBR1-BCL11A interaction (Fig. 3d). Since the p.L629* mutant is localized to the nucleus, similar to WT BCL11A protein, the lack of interaction with TBR1 cannot be attributed to aberrant localization of the variant (Supp. Figure 2b). Overall, our data indicate that the region spanning residues 629–773 in BCL11A is crucial for mediating the TBR1-BCL11A interaction.

### Effects of TBR1 variants on TBR1-BCL11A interaction

**Figure 4:**
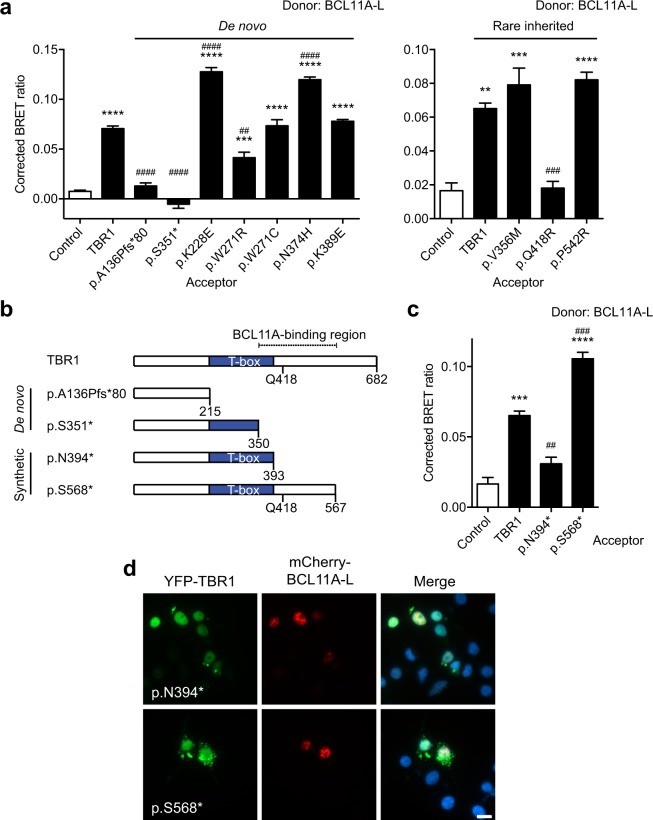
Effect of TBR1 variants on BCL11A-TBR1 interaction. (**a**) BRET assay for interaction between BCL11A-L and TBR1 variants. (**b**) Schematic representation of truncated TBR1 proteins. p.A136Pfs*80 and p.S351* are found in sporadic ASD cases. p.N394* and p.S568* are synthetic constructs. The glutamine at residue 418 (Q418) that may be important for the BCL11A-TBR1 interaction is also shown. (**c**) BRET assay for interaction between BCL11A-L and synthetic TBR1 variants. (**d**) Fluorescence micrographs of cells co-expressing BCL11A-L fused to mCherry (red; middle panel) and synthetic TBR1 variants fused to YFP (green; left panel). Nuclei were stained using Hoescht 33342 (blue). Scale bar = 10 µm. In (**a**) and (**c**) bars represent the corrected mean BRET ratio ± S.E.M. of one representative experiment performed in triplicate (**P < 0.01, ***P < 0.001, ****P < 0.0001 when compared to NLS-control, ^##^*P* < 0.01, ^###^*P* < 0.001, ^####^*P* < 0.0001 when compared to WT TBR1, one-way ANOVA and *post-hoc* Tukey’s test).

Our prior work demonstrated that TBR1 variants resulting from *de novo* truncating and missense mutations identified in sporadic ASD cases abolish the TBR1-FOXP2 interaction^18^. Using the BRET assay, we investigated the effects of these four TBR1 variants (p.A136Pfs*80, p.S351*, p.K228E, and p.N374H), as well as the three *de novo* TBR1 variants newly studied here (p.W271R, p.W271C and p.K389E), on the TBR1-BCL11A interaction (Fig. 1a). Overall, the *de novo* missense TBR1 variants p.K228E, p.W271R, p.W271C, p.N374H, and p.K389E did not disrupt the TBR1-BCL11A interaction (Fig. 4a and Supp. Figure 3a), although the BRET ratio in cells co-expressing YFP-p.W271R (acceptor) and *Renilla* luciferase-BCL11A (donor) was moderately decreased compared to WT TBR1 (Fig. 4a). This finding may be explained by the low expression levels observed for p.W271R (Fig. 1c,f and Supp. Figure 1a), which would reduce the availability of YFP-acceptor molecules.

The truncating variants of TBR1, p.A136Pfs*80 and p.S351*, which lack all or part of the T-box domain, were unable to interact with BCL11A (Fig. 4a and b). This lack of interaction cannot be explained by aberrant subcellular localization of the mutant TBR1 protein, as these variants localized both to the cytoplasm and nucleus, as previously reported (Supp. Figure 3a)^18^. It is possible that the C-terminal region in TBR1, including the T-box domain, is involved in the interaction.

To map the BCL11A binding site in TBR1, we performed BRET assays with two truncated TBR1 proteins (p.N394* and p.S568*) that have been described before^18^. The p.N394* variant is truncated just after the T-box domain, whereas p.S568* encodes a longer protein that includes part of the C-terminal region (Fig. 4b). Our assays indicated that although p.S568* did not disrupt the BCL11A-TBR1 interaction, the p.N394* variant displayed reduced interaction with BCL11A (Fig. 4c). This observation cannot be explained by altered subcellular localization of the variant, as it is found in both the cytoplasm and the nucleus, and co-localizes with nuclear BCL11A (Fig. 4d). It is therefore possible that the region encompassing residues 351–567 might be important for TBR1-BCL11A interaction (Fig. 4b). When we analyzed the effects of three rare inherited TBR1 variants that fall within this region - p.V356M, p.Q418R, and p.P542R^18^ - we found that the p.Q418R variant abolished the TBR1-BCL11A interaction, whereas the other variants did not disturb this interaction (Fig. 4a and Supp. Figure 3b). This finding is intriguing because p.Q418R has previously been reported to abolish the interaction between TBR1 and FOXP2^18^, suggesting that Q418 may be crucial for protein interactions.

## Discussion

In this study we examined the functional consequences of three *de novo* missense *TBR1* variants located in the T-box DNA-binding domain that were uncovered in sporadic ASD (Table 1). Two of these variants had severe effects on several aspects of TBR1 protein function. We found that the TBR1 variants abolished interactions with the FOXP1 and FOXP2 transcription factors involved in ID and severe speech/language disorder, respectively. We also show evidence for a novel interaction between TBR1 and BCL11A, a regulatory protein implicated in a neurodevelopmental syndrome characterized by developmental delay, ID and language deficits. Overall, our data strengthen the notion of molecular links between the etiology of ASD, ID and language-related disorders.

Of the three newly tested variants located in the T-box domain, two involve the tryptophan at position 271. Both variants, p.W271R and p.W271C, are predicted to be pathogenic by several computational tools, including CADD^29,35^. Moreover, our homology model of the T-box domain bound to DNA and mutation analysis suggests that both p.W271R and p.W271C would destabilize the core structure of the T-box, which could lead to misfolding of the protein. Indeed, we found that p.W271C had severe effects on protein function, disrupting localization and interactions with CASK, FOXP1, and FOXP2. In contrast, the p.W271R variant did not affect any of the aspects of protein function tested in our assays, although we did observe lower protein levels in cells expressing the variant protein. These findings have parallels in studies of missense variants implicated in other phenotypes; for example in cases of cancer, there are missense variants in *NF2* and *BRCA1* that do not appear to affect protein function, but reduce protein stability, and subsequently protein levels^36,37^. Thus, it is possible that reduced protein levels of the p.W271R variant of TBR1 in our study could contribute to the associated neurodevelopmental phenotype in the absence of observable functional effects on (for example) protein-protein interactions. The Grantham score, which takes into account the difference in composition, polarity and molecular volume of the residue, calculates that the physicochemical difference for p.W271R is moderate (101/215), while for p.W271C it is large (215/215)^38^. Indeed, our protein homology modeling suggests that the change from a tryptophan to a cysteine would leave a large opening in the T-box core structure in the case of p.W271C, whereas the change to an arginine, which is more comparable in size to a tryptophan, would not have such a big effect in the case of p.W271R. Therefore, the substitution of a cysteine at this position may have a stronger impact on TBR1 protein conformation than a substitution to an arginine, resulting in more pronounced functional effects.

Interestingly, all *de novo* missense variants tested here, including p.W271R, but none of the rare inherited TBR1 variants, showed increased protein stability compared to WT protein, which may result in an amplification of functional effects. Therefore, in the case of p.W271R, it may be that the increased protein stability contributes to the pathogenic profile. It is also possible that beyond altered protein stability, p.W271R confers additional, albeit subtle, effects *in vivo* that could not be detected in our cell-based experiments. Further analysis of this variant *in vivo*, focusing on neuronal circuitry and cortical development could be more informative in assessing its role in the etiology of ASD, although such experiments would be more laborious and expensive to carry out. *In vivo* investigations of a missense *TBR1* variant that is located in the T-box domain (p.N374H)^14^, but does not disturb transrepression activity in cell assays^18^, show that the variant results in reduced control of axonal growth and differentiation in mouse primary amygdalar neurons^7^.

Another possible explanation for the lack of significant functional effects in our cell-based assays may be that the p.W271R variant is actually a tolerated variant that does not contribute to the observed phenotype in the ASD case. Because the discovery of p.W271R came through whole-exome sequencing screens^15^, which mainly survey the protein-coding regions of the genome, it may be that an undetected variant in regulatory regions of non-coding DNA plays a role in the observed phenotype. Whole-genome sequencing, which surveys the entire genome for changes, is emerging as a more powerful tool for detecting causal variants^39^, especially as DNA sequencing costs continue to decrease.

The three T-box variants studied here did not affect the repression of a conserved element near *Fezf2*^3^, in line with observations for two previously assessed T-box variants in TBR1, p.K228E and p.N374H^18^, suggesting that these variants at least partially retain their ability to bind DNA. These findings are intriguing, as all four mutated residues are predicted to be either in direct contact with the DNA backbone (K228 and K389) or important for the stabilization of the T-box structure (W271 and N374), based on our protein homology model of the T-box domain-DNA complex. It is tempting to speculate that although the T-box in TBR1 is important for DNA-binding, activity domains may lie outside this region, as has been reported for other T-box proteins, such as Tbx3 and T element^40,41^.

In addition to evaluating the effects of *de novo* missense *TBR1* variants on protein function, we expanded the known TBR1 interactome by showing a novel protein-protein interaction with the BCL11A transcription factor. This interaction may be important for the regulation of downstream targets in the developing cortex, where these proteins co-localize^26^. Detailed characterization of the TBR1-BCL11A interaction indicates an extended BCL11A binding site in TBR1 between residues 351–567. Previous work has demonstrated that a similar region of the TBR1 protein, which also encompasses the C-terminus of the T-box domain, is important for interactions with CASK^8^ and with FOXP2^18^. Out of the *de novo* (p.N374H, p.K389E) and rare inherited (p.V356M, p.Q418R, p.P542R) missense variants that are located in the proposed binding region, only the p.Q418R variant abolished the TBR1-BCL11A interaction. This is in line with reported findings on the interaction between TBR1 and FOXP2^18^, suggesting that this residue is important for protein interactions. We also propose that residues 629–773 of BCL11A are sufficient for interaction with TBR1. Indeed, a similar region spanning residues 651–670 of BCL11A is important for interaction of BCL11A with NR2F1, NR2F2, NR2F6 and NR2E1^42^. Given that the residues 1–211 of the BCL11A protein mediate the BCL11A-CASK interaction^33^, it is unlikely that we detected an indirect interaction between TBR1 and BCL11A via CASK.

Our findings provide insight into shared molecular mechanisms underlying distinct neurodevelopmental disorders. Variants in *TBR1* and *BCL11A* result in phenotypes with notable overlaps, characterized by ID, ASD and speech and language problems^18,28,43^. Pathological truncating *TBR1* variants did not interact with BCL11A in our assays, pointing towards disrupted molecular networks. However, truncated variants of this kind are prone to nonsense mediated decay in the affected probands, and therefore may act as null alleles. In contrast, protein variants arising from pathogenic missense mutations in either protein did not disrupt the TBR1-BCL11A interaction. These findings suggest that the overlap in the phenotypes of patients carrying *TBR1* or *BCL11A* missense variants cannot be explained by a direct effect on the TBR1-BCL11A interaction. Nonetheless, such variants might potentially exert dominant negative effects by disrupting other aspects of TBR1 or BCL11A function, which could affect shared pathways, such as co-regulation of downstream targets. For example, *TBR1* missense variants are known to disrupt interactions with FOXP2^18^, whereas BCL11A variants affect homodimerization^28^.

Overall, our work underscores the importance of carrying out functional characterization of novel variants emerging from next-generation sequencing studies, even when the affected gene has been previously implicated in disorder. Combined together, sequencing screens, functional studies and detailed phenotypic investigations, will be paramount in furthering our understanding of the molecular mechanisms that go awry in neurodevelopmental disorders.

## Methods

### Cell culture and transfection

HEK293 cells (85120602, ECACC) were cultured in DMEM supplemented with 10% fetal bovine serum (both Invitrogen) at 37 °C with 5% CO_2_. Transfections were performed using GeneJuice (Millipore) following the manufacturer’s protocol.

### DNA expression constructs and site-directed mutagenesis

The cloning of TBR1 (NM_006593), BCL11A-L (NM_018014), BCL11A-S (NM_138559), CASK (XM_011543993), FOXP2 (NM_014491) and FOXP1 (NM_032682) has been described previously^18,28,44^. Variants in TBR1 and BCL11A-L were generated using the QuikChange Lightning Site-Directed Mutagenesis Kit (Agilent). The primers used for site-directed mutagenesis are listed in Supplementary Table 1 and^18,28^. BCL11A-L N-terminal deletions (Ndel1 and Ndel2) were amplified from BCL11A-L and ligated into pCR2.1-TOPO vector (Invitrogen). The primers used to generate the N-terminal deletions are listed in Supplementary Table 2. cDNAs were subcloned into pRluc, pYFP and a modified pmCherry-C1 vector (Clontech) using *EcoRI*/*XbaI* (TBR1 variants), *EcoRI*/*KpnI* (CASK) and *BamHI*/*XbaI* (BCL11A, FOXP2, FOXP1) restriction sites. All constructs were verified by Sanger sequencing.

### Three-dimensional modeling

The protein structure of the TBR1 T-box in interaction with DNA was modeled using the homology modeling script in the WHAT IF^45^ & YASARA^46^ Twinset with standard parameters. The PDB file 2X6U^47^ which contains the human TBX5 T-box domain (51.6% sequence identity with the TBR1 T-box domain) served as a template for the protein structure. The DNA was taken from PDB file 1XBR^48^.

### Western blotting

Whole-cell lysates were collected 48 h post-transfection by treatment with lysis buffer (100 mm Tris pH 7.5, 150 mM NaCl, 10 mM EDTA, 0.2% Triton X-100, 1% PMSF, protease inhibitor cocktail; all from Sigma–Aldrich). Cells were lysed for 10 min at 4 °C followed by centrifugation for 30 min at 10,000 g at 4 °C. Proteins were resolved on 4–15% Mini-PROTEAN TGX Precast Gels (Bio-Rad) and transferred onto polyvinylidene fluoride membranes. Membranes were blocked in 1–5% milk for 1 h at room temperature and then probed with mouse-anti-EGFP (for pYFP constructs; 1:8000; Clontech) or rabbit-anti-Rluc antibody (for pRluc constructs; 1:2000; GeneTex) for 2 h at room temperature. Next, membranes were incubated with HRP-conjugated goat-anti-mouse (1:2000; Bio-Rad) or donkey-anti-rabbit antibody (1:2000; Abcam) for 1 h at room temperature. Bands were visualized with Novex ECL Chemiluminescent Substrate Reagent Kit (Invitrogen) using a ChemiDoc XRS + System (Bio-Rad). Equal protein loading was confirmed by stripping and reprobing with mouse-anti-β-actin antibody (1:10,000; Sigma).

### Fluorescence-based quantification of protein expression levels

Cells were transfected in triplicate in clear-bottomed black 96-well plates with YFP-tagged TBR1 variants, as well as a modified pmCherry-C1 plasmid to normalize for transfection efficiency. After 48 h, fluorescence intensities of YFP (Ex: 505 nm, Em: 545 nm) and mCherry (Ex: 580 nm, Em: 620 nm) were measured at the Infinite M200PRO microplate reader (Tecan). Relative fluorescence intensities were calculated by dividing the background-subtracted YFP values by the background-subtracted mCherry values. The experiment was repeated using mCherry-tagged TBR1 variants, with a pYFP plasmid as the normalizer.

### Fluorescence-based quantification of protein degradation

Cells were transfected in triplicate in clear-bottomed black 96-well plates with YFP-tagged TBR1 variants. After 24 h, cycloheximide (Sigma) was added at a final concentration of 50 µg/ml. Cells were incubated at 37 °C with 5% CO_2_ in the Infinite M200PRO microplate reader (Tecan), and the fluorescence intensity of YFP (Ex: 505 nm, Em: 545 nm) was measured at 0, 2, 4, 6 and 8 h after administration of cycloheximide.

### Fluorescence imaging

Cells were grown on coverslips coated with poly-L-lysine (Sigma-Aldrich). 48 h post-transfection, cells were fixed with 4% paraformaldehyde (Electron Microscopy Sciences). Nuclei were stained with Hoescht 33342 (Invitrogen). Fluorescence images were acquired with an Axiovert A-1 fluorescent microscope and ZEN Image Software (Zeiss). For quantitative microscopy, two (for YFP-TBR1 nuclear aggregation) or four (for CASK and mCherry-TBR1 nuclear aggregation when co-expressed with YFP-TBR1 variants) 3 × 4 stitched images were taken with a 40x objective for each experiment and manually counted using ImageJ software. Quantification was conducted with the experimenter blinded to the conditions.

### Luciferase reporter assay

Luciferase reporter assays were performed with a pGL3-CMV firefly luciferase reporter plasmid containing a Tbr1-binding site near *Fezf2* as described previously^3,18^. Cells were transfected with 45 ng of firefly luciferase reporter construct, 5 ng of *Renilla* luciferase (Rluc) normalization control (pRL-TK; Promega) and 200 ng TBR1 expression construct (WT or variant in pYFP) or empty vector (pYFP). After 48 h, firefly luciferase and Rluc activity was measured using the Dual-Luciferase Reporter Assay system (Promega) at the Infinite F200PRO Microplate reader (Tecan).

### BRET assay

BRET assays were performed as previously described^32^. Cells were transfected in white clear-bottomed 96-well plates with equimolar concentrations of YFP and Rluc fusion proteins. YFP and Rluc fused to a C-terminal NLS were used as control proteins. After 48 h, medium was replaced with phenol red-free DMEM, supplemented with 10% fetal bovine serum (both Invitrogen), containing 60 µM EnduRen Live Cell Substrate (Promega). After incubation for 4 h at 37 °C, measurements were taken in live cells with an Infinite F200PRO Microplate reader (Tecan) using the Blue1 and Green1 filters. Corrected BRET ratios were calculated with the following formula: [Green1_(experimental condition)_/Blue1_(experimental condition)_] − [Green1_(control condition)_/Blue1_(control condition)_], with only the Rluc control protein expressed in the control condition. YFP fluorescence was measured separately (Ex: 505 nm, Em: 545 nm) to quantify expression of the YFP-fusion proteins.

### Statistical analysis

For protein expression experiments, quantified microscopy data, luciferase reporter assays and BRET assays, statistical analysis was done using a one-way or two-way ANOVA followed by a Tukey’s *post hoc* test. For protein degradation assays, statistical analysis was done using a repeated measures two-way ANOVA followed by a Tukey’s *post hoc* test. The analyses were performed with GraphPad Prism Software.

## Electronic supplementary material

Supplementary Information

## Data Availability

All datasets generated and analyzed during the current study are available from the corresponding authors (P.D. and S.E.F.) on request.

## References

[1] Bulfone A (1995). T-brain-1: a homolog of Brachyury whose expression defines molecularly distinct domains within the cerebral cortex. Neuron.

[2] Bedogni F (2010). Tbr1 regulates regional and laminar identity of postmitotic neurons in developing neocortex. Proc Natl Acad Sci USA.

[3] Han W (2011). TBR1 directly represses Fezf2 to control the laminar origin and development of the corticospinal tract. Proc Natl Acad Sci USA.

[4] McKenna WL (2011). Tbr1 and Fezf2 regulate alternate corticofugal neuronal identities during neocortical development. J Neurosci.

[5] Bulfone A (1998). An olfactory sensory map develops in the absence of normal projection neurons or GABAergic interneurons. Neuron.

[6] Hevner RF (2001). Tbr1 regulates differentiation of the preplate and layer 6. Neuron.

[7] Huang TN (2014). Tbr1 haploinsufficiency impairs amygdalar axonal projections and results in cognitive abnormality. Nat Neurosci.

[8] Hsueh YP, Wang TF, Yang FC, Sheng M (2000). Nuclear translocation and transcription regulation by the membrane-associated guanylate kinase CASK/LIN-2. Nature.

[9] Chuang HC, Huang TN, Hsueh YP (2014). Neuronal excitation upregulates Tbr1, a high-confidence risk gene of autism, mediating Grin2b expression in the adult brain. Front Cellular Neurosci.

[10] Notwell JH (2016). TBR1 regulates autism risk genes in the developing neocortex. Genome Res.

[11] Traylor RN (2012). Investigation of TBR1 Hemizygosity: Four Individuals with 2q24 Microdeletions. Mol Syndromol.

[12] Palumbo O (2014). TBR1 is the candidate gene for intellectual disability in patients with a 2q24.2 interstitial deletion. Am J Med Genet A.

[13] O’Roak BJ (2012). Multiplex targeted sequencing identifies recurrently mutated genes in autism spectrum disorders. Science.

[14] Neale BM (2012). Patterns and rates of exonic de novo mutations in autism spectrum disorders. Nature.

[15] Hamdan FF (2014). De novo mutations in moderate or severe intellectual disability. PLoS Genet.

[16] De Rubeis S (2014). Synaptic, transcriptional and chromatin genes disrupted in autism. Nature.

[17] O’Roak BJ (2014). Recurrent de novo mutations implicate novel genes underlying simplex autism risk. Nat Commun.

[18] Deriziotis P (2014). De novo TBR1 mutations in sporadic autism disrupt protein functions. Nat Commun.

[19] McDermott J.H., Study D.D.D., Clayton-Smith J., Briggs T.A. (2018). The TBR1 -related autistic-spectrum-disorder phenotype and its clinical spectrum. European Journal of Medical Genetics.

[20] Sollis E (2017). Equivalent missense variant in the FOXP2 and FOXP1 transcription factors causes distinct neurodevelopmental disorders. Hum Mutat.

[21] Lai CS, Fisher SE, Hurst JA, Vargha-Khadem F, Monaco AP (2001). A forkhead-domain gene is mutated in a severe speech and language disorder. Nature.

[22] Le Fevre AK (2013). FOXP1 mutations cause intellectual disability and a recognizable phenotype. Am J Med Genet A.

[23] Sollis E (2016). Identification and functional characterization of de novo FOXP1 variants provides novel insights into the etiology of neurodevelopmental disorder. Hum Mol Genet.

[24] Najm J (2008). Mutations of CASK cause an X-linked brain malformation phenotype with microcephaly and hypoplasia of the brainstem and cerebellum. Nat Genet.

[25] Sanders SJ (2012). De novo mutations revealed by whole-exome sequencing are strongly associated with autism. Nature.

[26] Canovas J (2015). The Specification of Cortical Subcerebral Projection Neurons Depends on the Direct Repression of TBR1 by CTIP1/BCL11a. J Neurosci.

[27] Woodworth MB (2016). Ctip1 Regulates the Balance between Specification of Distinct Projection Neuron Subtypes in Deep Cortical Layers. Cell Rep.

[28] Dias C (2016). BCL11A Haploinsufficiency Causes an Intellectual Disability Syndrome and Dysregulates Transcription. Am J Hum Genet.

[29] Kircher M (2014). A general framework for estimating the relative pathogenicity of human genetic variants. Nat Genet.

[30] Hsueh YP (2009). Calcium/calmodulin-dependent serine protein kinase and mental retardation. Annals Neurol.

[31] Wang GS (2004). Transcriptional modification by a CASK-interacting nucleosome assembly protein. Neuron.

[32] Deriziotis P, Graham SA, Estruch SB, Fisher SE (2014). Investigating protein-protein interactions in live cells using bioluminescence resonance energy transfer. J Vis Exp.

[33] Kuo TY, Hong CJ, Chien HL, Hsueh YP (2010). X-linked mental retardation gene CASK interacts with Bcl11A/CTIP1 and regulates axon branching and outgrowth. J Neurosci Res.

[34] Satterwhite E (2001). The BCL11 gene family: involvement of BCL11A in lymphoid malignancies. Blood.

[35] Adzhubei IA (2010). A method and server for predicting damaging missense mutations. Nat Methods.

[36] Yang C (2011). Missense mutations in the NF2 gene result in the quantitative loss of merlin protein and minimally affect protein intrinsic function. Proc Natl Acad Sci USA.

[37] Gaboriau DC, Rowling PJ, Morrison CG, Itzhaki LS (2015). Protein stability versus function: effects of destabilizing missense mutations on BRCA1 DNA repair activity. Biochem J.

[38] Grantham R (1974). Amino acid difference formula to help explain protein evolution. Science.

[39] Gilissen C (2014). Genome sequencing identifies major causes of severe intellectual disability. Nature.

[40] Carlson H, Ota S, Campbell CE, Hurlin PJ (2001). A dominant repression domain in Tbx3 mediates transcriptional repression and cell immortalization: relevance to mutations in Tbx3 that cause ulnar-mammary syndrome. Hum Mol Genet.

[41] Kispert A, Koschorz B, Herrmann BG (1995). The T protein encoded by Brachyury is a tissue-specific transcription factor. EMBO J.

[42] Chan CM (2013). A signature motif mediating selective interactions of BCL11A with the NR2E/F subfamily of orphan nuclear receptors. Nucleic Acids Res.

[43] Peter B, Matsushita M, Oda K, Raskind W (2014). De novo microdeletion of BCL11A is associated with severe speech sound disorder. Am J Med Genet A.

[44] Vernes SC (2006). Functional genetic analysis of mutations implicated in a human speech and language disorder. Hum Mol Genet.

[45] Vriend G. (1990). WHAT IF: A molecular modeling and drug design program. Journal of Molecular Graphics.

[46] Krieger E, Koraimann G, Vriend G (2002). Increasing the precision of comparative models with YASARA NOVA–a self-parameterizing force field. Proteins.

[47] Stirnimann CU, Ptchelkine D, Grimm C, Muller CW (2010). Structural basis of TBX5-DNA recognition: the T-box domain in its DNA-bound and -unbound form. J Mol Biol.

[48] Muller CW, Herrmann BG (1997). Crystallographic structure of the T domain-DNA complex of the Brachyury transcription factor. Nature.

[49] Kosugi S, Hasebe M, Tomita M, Yanagawa H (2009). Systematic identification of cell cycle-dependent yeast nucleocytoplasmic shuttling proteins by prediction of composite motifs. Proc Natl Acad Sci USA.

