# Supplementary material for: Functional characterization of TBR1 variants in neurodevelopmental disorder
Source: Sci Rep. 2018 Sep 24;8:14279. doi: 10.1038/s41598-018-32053-6 (PMC6155134; doi:10.1038/s41598-018-32053-6)
Supplement: Supplementary file 1 — Supplementary Information [file 41598_2018_32053_MOESM1_ESM.pdf]

Functional characterization of TBR1 variants in neurodevelopmental disorder.

Joery den Hoed[1], Elliot Sollis[1], Hanka Venselaar[2], Sara B. Estruch[1], Pelagia Deriziotis[1†*] and Simon E. Fisher[1,3†*]

[1]Language and Genetics Department, Max Planck Institute for Psycholinguistics, 6525 XD Nijmegen, The Netherlands

[2]Centre for Molecular and Biomolecular Informatics, Radboud University Nijmegen Medical Centre, 6525 GA Nijmegen, the Netherlands

[3]Donders Institute for Brain, Cognition and Behaviour, 6525 EN Nijmegen, The Netherlands

[†] these authors contributed equally

[*] to whom correspondence should be sent; pelagia.derizioti@mpi.nl; simon.fisher@mpi.nl

Supplementary Notes

TBR1 T-box protein modeling

We modeled the protein structure of the TBR1 T-box in interaction with the DNA using the homology modeling script in the WHAT IF[1] & YASARA[2] Twinset with standard parameters. As a template, we used PDB file 2X6U[3] which contains the human TBX5 T-box domain. The DNA was taken from PDB file 1XBR[4]. We performed mutation analysis on the *de novo TBR1* missense variants p.W271R, p.W271C and p.K389E, and on the previously studied missense variants p.K228E and p.N374H[5] (Figure 1).

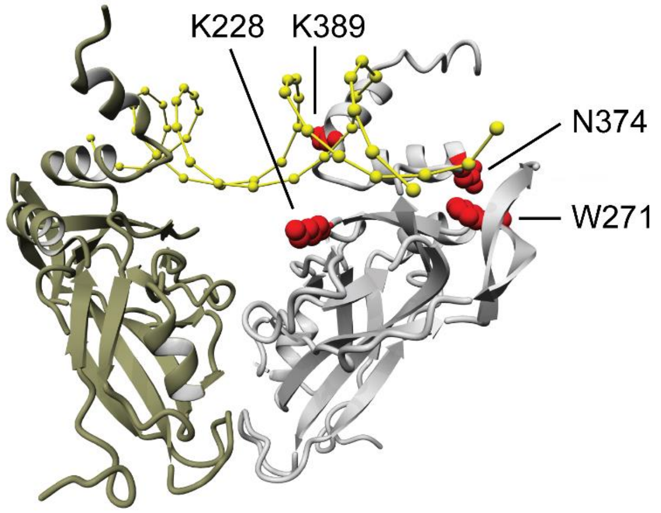

Figure 1. Overview of the TBR1 T-box domain model in interaction with DNA. The model was built as a dimer. Monomers are shown in gold and grey, and the DNA is depicted in yellow. The studied residues are depicted as red balls.

Mutation analysis

K228

K288 is a positively charged amino acid, located in close proximity to the negatively charged DNA phosphate backbone. This residue might be crucial for non-specific binding of the protein to the minor groove of the DNA.

A change to a glutamate (p.K228E) would change the charge of this residue from positive to negative, disrupting the ionic interaction with the DNA backbone (Figure 2).

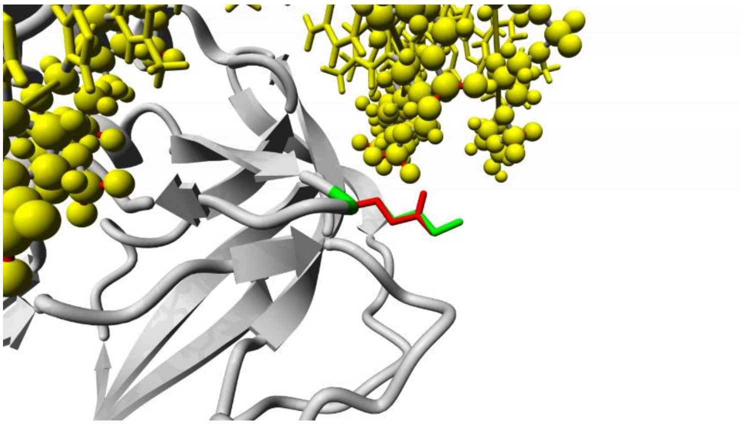

Figure 2. Close-up of the p.K228E variant. The TBR1 monomer is colored grey and the complete DNA molecule is shown in yellow. Phosphates in the DNA backbone are presented as balls, while the bases are depicted as sticks. The wild-type (K) and mutant (E) sidechains are shown in green and red respectively.

W271

W271 is located in the core of the T-box and is in contact with hydrophobic amino acids that surround this residue. This stabilizes the protein T-box domain. Therefore, this amino acid is indirectly important for DNA binding, by making sure that the DNA binding residues are positioned correctly.

A change from the big tryptophan sidechain to the much smaller cysteine (p.W271C) would leave an empty space in the core of the complex, which will destabilize the core of the T-box (Figure 3). Contacts with hydrophobic amino acids that stabilize the structure would be lost, which will affect the folding of the domain and the position of the DNA binding residues. Furthermore, cysteines can form disulfide bonds, and a free cysteine sidechain might therefore be able to bind to other mutated proteins, which could result in aggregation.

A change of W271 to an arginine (p.W271R) would also disrupt the stabilizing hydrophobic contacts in the core of the domain, albeit to a lesser extent because arginine is also a large amino acid (Figure 3). Still, many hydrophobic contacts will be lost and this might cause destabilization of the domain and affect the DNA binding. The effect of the introduction of the positive charge in the arginine's sidechain is unclear.

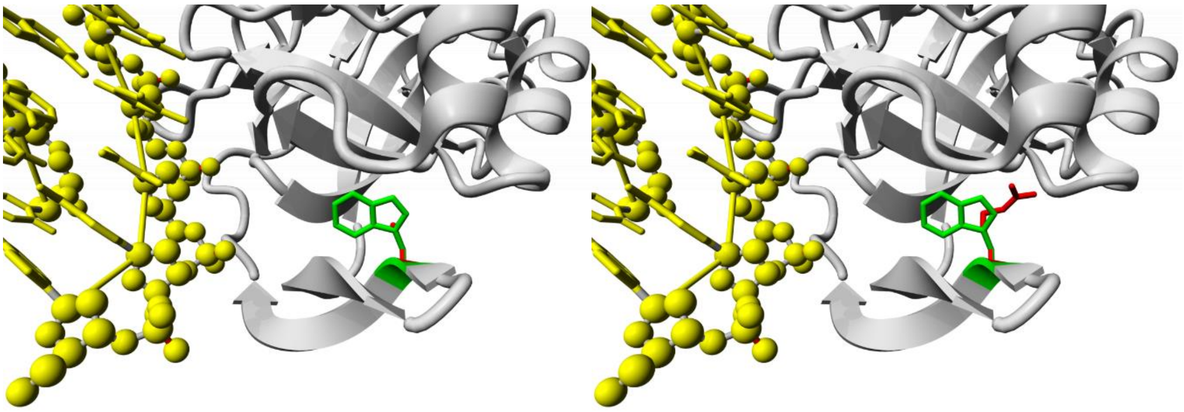

Figure 3. Close-up of the p.W271C (left) and p.W271R (right) variants, colored as in Figure 1.

N374

N374 is located in the T-box structure, forming hydrogen bonds with surrounding residues, thereby stabilizing the protein domain. Similar to the previous W271, this amino acid is indirectly important for DNA binding by making sure that the DNA binding residues are positioned correctly.

A change to a histidine (p.N374H) prevents the formation of some of the hydrogen bonds, which may affect the stability of the domain (Figure 4).

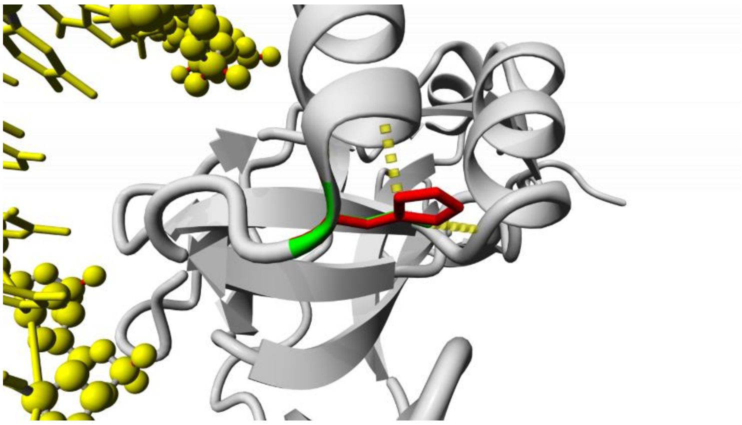

Figure 4. Close-up of the p.N374H mutation, colored as in Figure 1. Yellow dotted lines indicate the hydrogen bonds formed by the wild-type asparagine.

K389

K389 is a positively charged amino acid in close proximity to the negatively charged backbone of the DNA. The residue might be crucial for non-specific binding of the protein to the minor groove of the DNA.

A change to a glutamate (p.K389E) would change the charge of this residue from positive to negative, thereby disrupting the binding to the negatively charged phosphates in the DNA backbone (Figure 5).

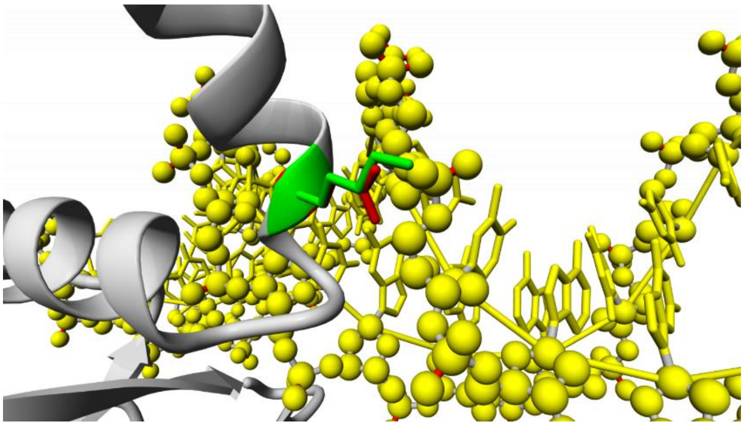

Figure 5. Close-up of the p.K389E mutation, colored as in Figure 1.

Homodimerization

In the templates used for our homology model of the TBR1 T-box domain, the protein is presented as a dimer (Figure 1). In the model assembly created by YASARA, all the models

are either monomers or in this particular dimer conformation. The main interacting residues for dimerization in this dimer conformation are 291-295 and 345-348. However, no strong ionic or hydrophobic interactions could be identified.

Therefore, we also studied other dimer conformations (Figure 6). We analyzed T-box domain conformations with >50% sequence identity with our TBR1 sequence, obtained from PDB files 4S0H[6] (Figure 6a), 5T1J[7] (Figure 6b), 5BQD[6] (Figure 6c). We superposed the dimers in these files with our homology model and studied the resulting position of the second monomer. In these other dimer conformations the main interactions between the monomers are made using the residues in the β-strands distal to the DNA binding side. The residues K228, W271, N374 and K389 are not located at the dimer interfaces in any of the dimer conformations, suggesting that they do not play a direct role in homodimerization.

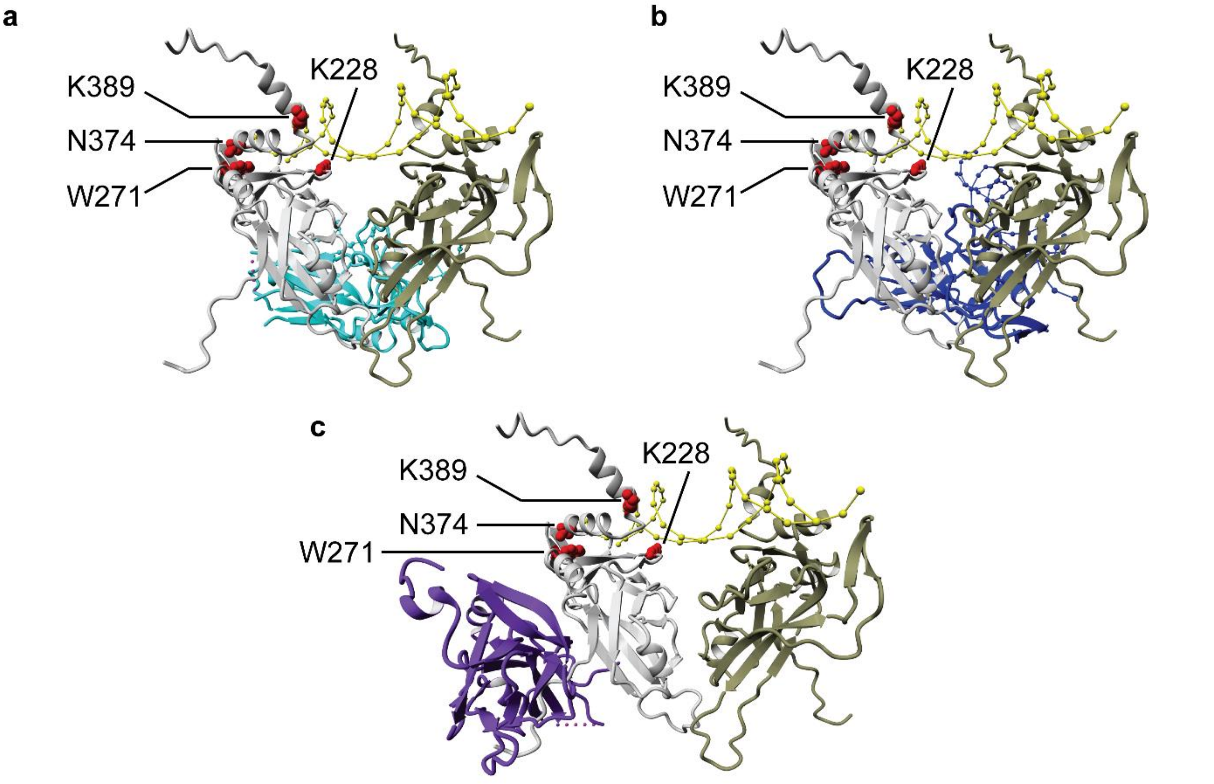

Figure 6. Superposition of other TBR1 dimer conformations. a) Superposition assembly for PDB file 4S0H (turquois) based on one of the monomers of our homology model depicted in light grey. **b**) Superposition assembly for PDB file 5T1J (blue) based on one of the monomers of our homology model depicted in light grey. **c**) Superposition assembly for PDB file 5BQD (purple) based on one of the monomers of our homology model depicted in light grey. In **a**, **b**, and **c** the DNA is shown in yellow and the studied residues (K228, W271, N374 and K389) are presented as red balls. Note that none of the mutated residues studied in this paper are close to a dimer surface in any of these conformations.

References

1 Vriend, G. WHAT IF: a molecular modeling and drug design program. *Journal of molecular graphics* **8**, 52-56, 29 (1990).

2 Krieger, E., Koraimann, G. & Vriend, G. Increasing the precision of comparative models with YASARA NOVA--a self-parameterizing force field. *Proteins* **47**, 393-402 (2002).

3 Stirnimann, C. U., Ptchelkine, D., Grimm, C. & Muller, C. W. Structural basis of TBX5-DNA recognition: the T-box domain in its DNA-bound and -unbound form. *Journal of molecular biology* **400**, 71-81, doi:10.1016/j.jmb.2010.04.052 (2010).

4 Muller, C. W. & Herrmann, B. G. Crystallographic structure of the T domain-DNA complex of the Brachyury transcription factor. *Nature* **389**, 884-888, doi:10.1038/39929 (1997).

5 Deriziotis, P. *et al.* De novo TBR1 mutations in sporadic autism disrupt protein functions. *Nature communications* **5**, 4954, doi:10.1038/ncomms5954 (2014).

6 Pradhan, L. *et al.* Intermolecular Interactions of Cardiac Transcription Factors NKX2.5 and TBX5. *Biochemistry* **55**, 1702-1710, doi:10.1021/acs.biochem.6b00171 (2016).

7 Liu, C. F. *et al.* Crystal structure of the DNA binding domain of the transcription factor T-bet suggests simultaneous recognition of distant genome sites. *Proc Natl Acad Sci U S A* **113**, E6572-e6581, doi:10.1073/pnas.1613914113 (2016).

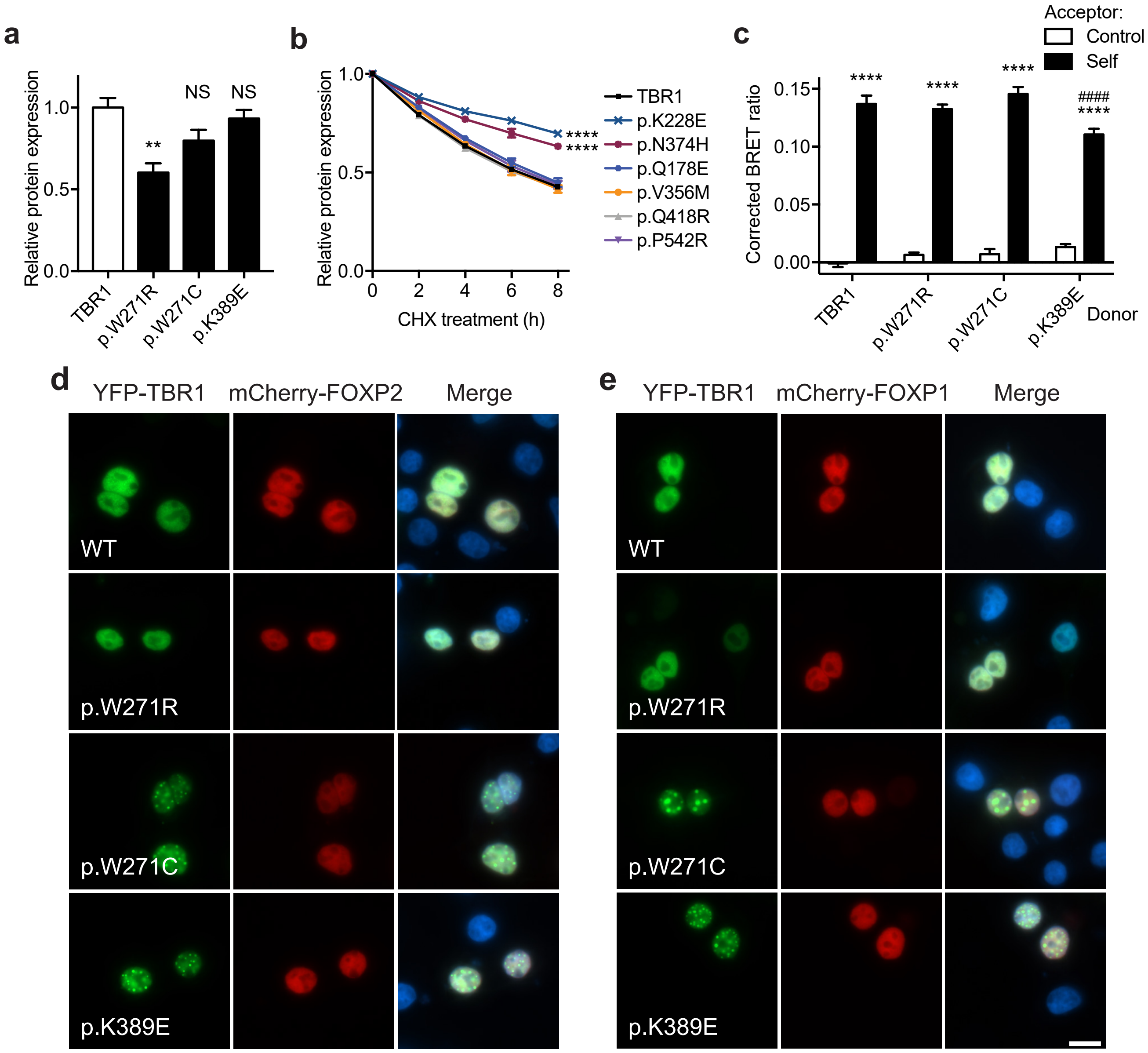

Supplementary Figure 1. Functional characterization of *de novo* missense *TBR1* variants. a) Relative expression levels of TBR1 protein variants in live cells as measured by mCherry fluorescence (mean of three independent experiments performed in triplicate ± S.E.M., $^{**}P<0.01$, NS: not significant, one-way ANOVA and *post-hoc* Tukey's test). **b)** Assay for protein degradation of TBR1 variants using cycloheximide (CHX) to arrest protein synthesis. Values represent the mean relative expression levels of TBR1 protein variants in live cells as measured by YFP fluorescence ± S.E.M. of three independent experiments performed in triplicate ($^{****}P<0.0001$ at 8 h time point compared to WT TBR1, repeated measures two-way ANOVA and *post-hoc* Tukey's test). **c)** BRET assay for self-association of TBR1 variants in live cells. Bars represent the corrected mean BRET ratios ± S.E.M. of one representative experiment performed in triplicate ($^{****}P<0.0001$ when compared to NLS-control, $^{\#\#\#\#}P<0.0001$ when compared to WT TBR1, two-way ANOVA and *post-hoc* Tukey's test). **d)** Fluorescence micrographs of cells co-transfected with TBR1 variants and FOXP2. TBR1 variants are fused to YFP (green; left panel) and FOXP2 is fused to mCherry (red; middle panel). **e)** Fluorescence micrographs of cells co-transfected with TBR1 variants and FOXP1. TBR1 variants are fused to YFP (green, left panel) and FOXP1 is fused to mCherry (red, middle panel). In **d** and **e** nuclei were stained with Hoescht 33342 (blue). Scale bar = 10 µm.

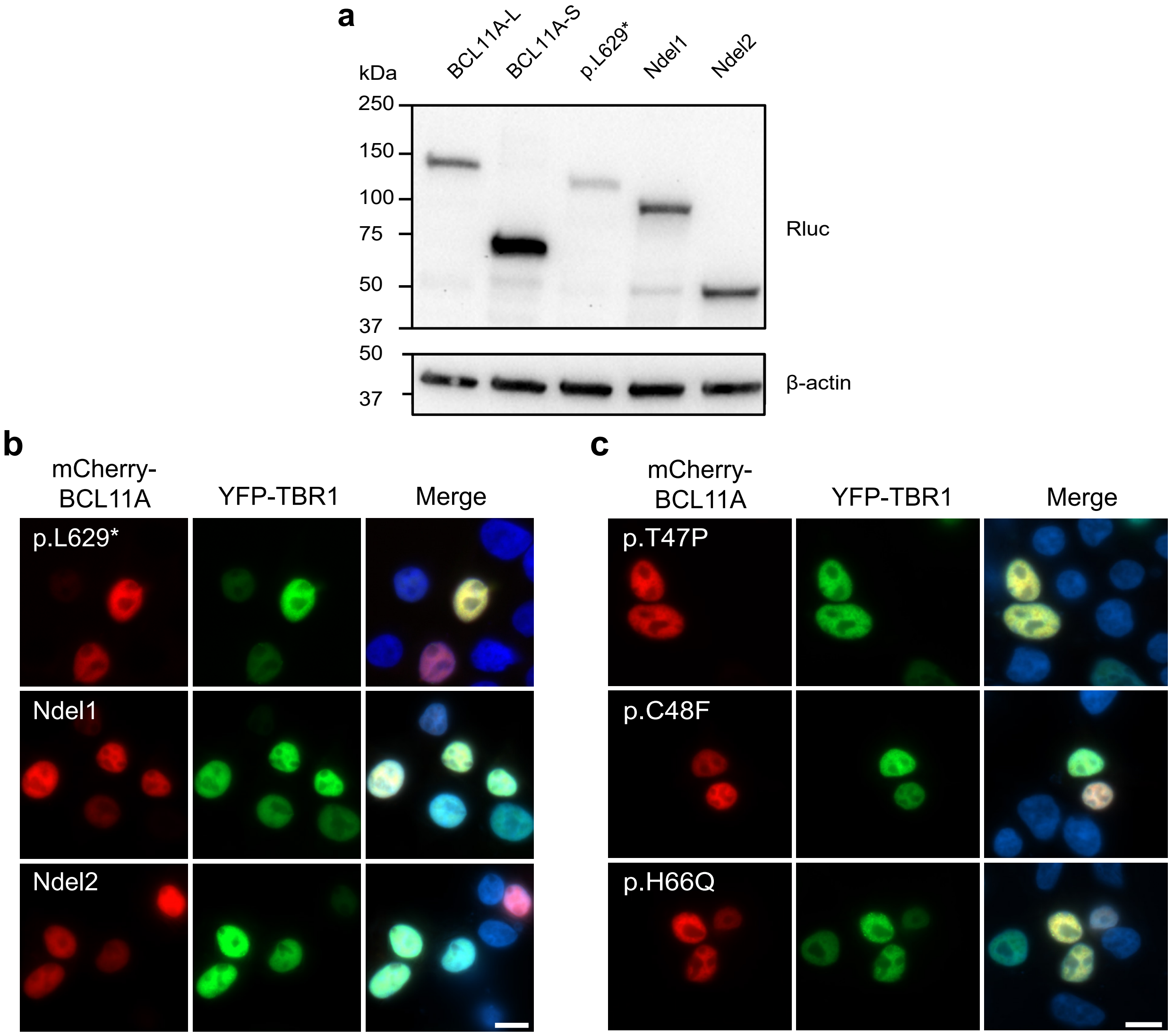

Supplementary Figure 2. TBR1 interacts with BCL11A-L. a) Immunoblot of whole-cell lysates expressing Rluc-tagged BCL11A synthetic variants probed with anti-Rluc antibody. Expected molecular weight for Rluc-BCL11A proteins: ~123 kDa = BCL11A-L; ~66 kDa = BCL11A-S; ~108 kDa = L629*; ~96 kDa = Ndel1; ~54 kDa = Ndel2. The blot was stripped and re-probed for β-actin, to ensure equal protein loading. **b**) Fluorescence micrographs of cells co-expressing BCL11A synthetic variants fused to mCherry (red; left panel) and WT TBR1 fused to YFP (green; middle panel). **c**) Fluorescence micrographs of cells co-expressing *de novo* BCL11A variants found in cases of Dias-Logan syndrome fused to mCherry (red; left panel) and WT TBR1 fused to YFP (green; middle panel). In **b** and **c** nuclei were stained with Hoescht 33342 (blue). Scale bar = 10 µm.

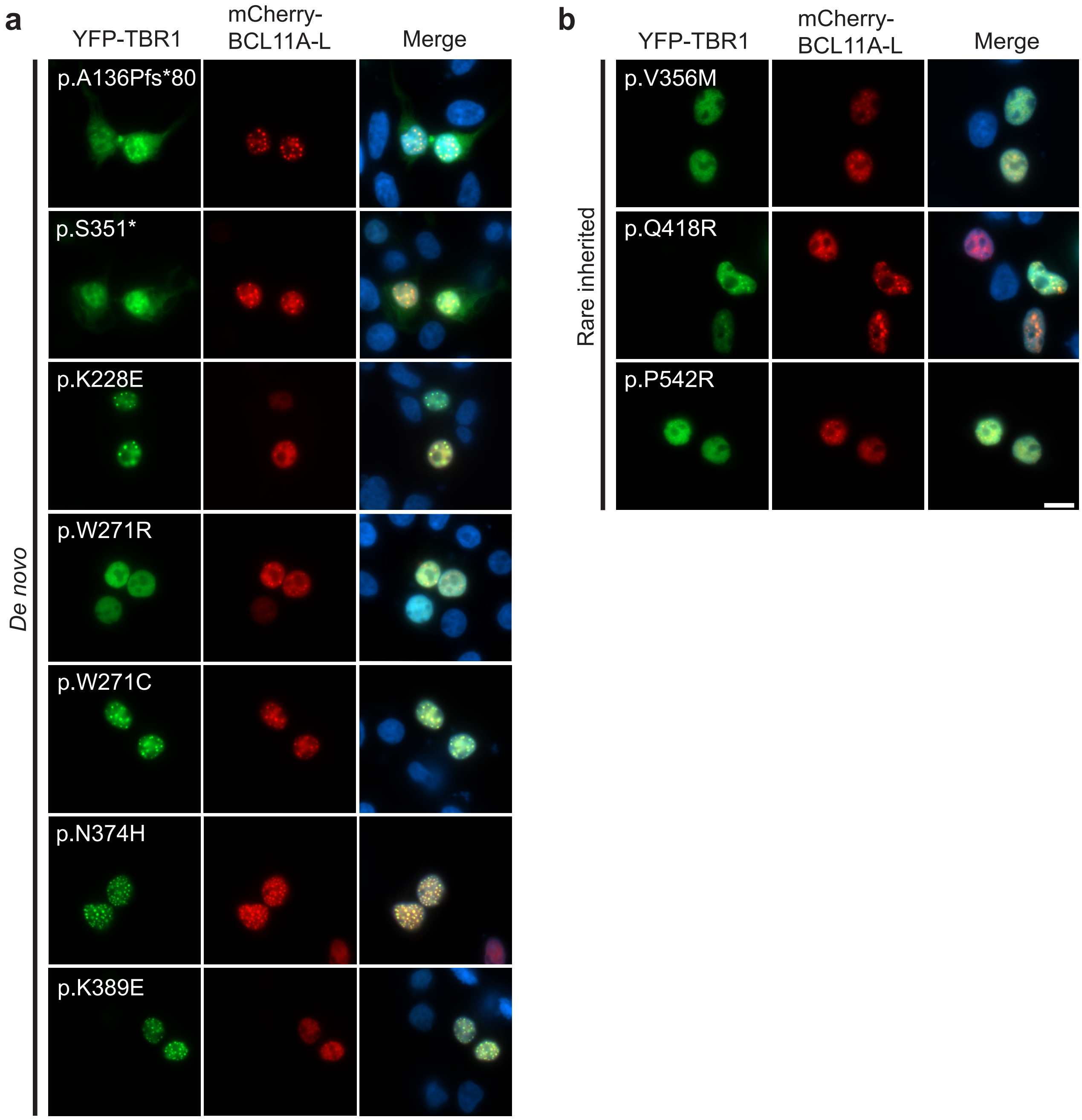

Supplementary Figure 3. Effect of TBR1 variants on BCL11A-TBR1 interaction. a) Fluorescence micrographs of cells co-expressing *de novo* TBR1 variants found in cases of sporadic ASD fused to YFP (green; left panel) and WT BCL11A-L fused to mCherry (red; middle panel). **b**) Fluorescence micrographs of cells co-expressing rare inherited TBR1 variants found in cases of sporadic ASD fused to YFP (green; left panel) and WT BCL11A-L fused to mCherry (red; middle panel). In **a** and **b** nuclei were stained with Hoescht 33342 (blue). Scale bar = 10 µm.

Supplementary Table 1. Primers used for site-directed mutagenesis.

cDNA	Variant	Forward primer (5'-3')	Reverse primer (5'-3')
TBR1	p.W271R	TGGAGGTTTCAAGGAGGCAAA CGGGTTCCTTGC	GCAAGGAACCCGTTTGCCTCC TTGAAACCTCCA
	p.W271C	TTTCAAGGAGGCAAATGTGTTC CTTGCGGCAAAG	CTTTGCCGCAAGGAACACATTT GCCTCCTTGAAA
	p.K389E	AAATAGATCACAACCCTTTTGC AGAAGGATTTCGGGATAATTAT GAC	GTCATAATTATCCCGAAATCCT TCTGCAAAAGGGTTGTGATCTA TTT
BCL11A-L	p.L629*	CAGCCCCAGCTCGCCGAAGAA GAAGCGCAAGGTCTAACTGAG CCCCTTCT	AGAAGGGGCTCAGTTAGACCT TGCGCTTCTTCTTCGGCGAGC TGGGGCTG

Supplementary Table 2. Primers used for generating BCL11A-L N-terminal deletions.

cDNA	Variant	Forward primer (5'-3')	Reverse primer (5'-3')
BCL11A-L	Ndel1	GGATCCTACCAGGATCAGTAT CGAGAGA	GCTAGCTCAGAACTTAAGGGC TCTCG
	Ndel2	GGATCCTACTGAGCCCCTTCT CTAAGCG	GCTAGCTCAGAACTTAAGGGC TCTCG
